# Measles outbreak in Romania: understanding factors related to suboptimal vaccination uptake

**DOI:** 10.1093/eurpub/ckaa079

**Published:** 2020-05-27

**Authors:** Katrine Bach Habersaat, Adriana Pistol, Aurora Stanescu, Catherine Hewitt, Miljana Grbic, Cassandra Butu, Cath Jackson

**Affiliations:** c1 Vaccine-preventable Diseases and Immunization, World Health Organization (WHO) Regional Office for Europe, Copenhagen, Denmark; c2 National Center for Communicable Diseases Surveillance and Control, National Institute of Public Health, Bucharest, Romania; c3 York Trials Unit, Department of Health Sciences, University of York, York, UK; c4 WHO Country Office, Bucharest, Romania; c5 Valid Research Ltd, Wetherby, UK

## Abstract

**Background:**

A large measles outbreak started in Romania in 2016. Current study aimed to (i) clarify who was affected by the outbreak, (ii) identify their barriers and drivers to vaccination and (iii) explore variation by population group.

**Methods:**

This was a two-component study. Outbreak surveillance data for 6743 measles cases were reviewed to identify key characteristics. A survey was administered via telephone to 704 caregivers of measles cases (520 respondents) to explore capability, opportunity and motivation barriers to vaccination. Data were summarized descriptively for respondent characteristics and statements. Differences by population group (education, household income, ethnicity, setting and mobility) were explored using *χ*^2^ tests, Fisher’s exact tests or regression models.

**Results:**

Most cases were unvaccinated and lived in low coverage areas. Ethnic minorities were disproportionally affected. Most caregivers felt welcome at health facilities. Some were less satisfied with the waiting time and had found the vaccine out of stock. Not everybody knew that vaccines were free of charge. Less than half knew the child’s next vaccination date, some had not been informed and did not know where to seek this information. Some said their peers did not vaccinate. Beliefs were generally supportive of vaccination; but many were concerned about vaccine safety and found they had not received good information about this. Conclusions varied greatly between minorities and less educated groups, compared with people with higher education levels.

**Conclusions:**

Identifying characteristics of the population affected and underlying factors can inform a strategy to avoid future outbreaks and further research to obtain deeper insights.

## Introduction 

Large measles outbreaks in the European Region, with more than 100 000 cases in 2019,^1^ are occurring after years where countries have failed to reach the 95% coverage with two doses of measles vaccine at national and subnational levels necessary to eliminate the disease.^2^ Tailored, innovative strategies are critical in reaching sub-optimally vaccinated population groups.^3^^,^^4^ On this background, the World Health Organization (WHO) Regional Office for Europe supports its Member States in exploring determinants of vaccination to inform interventions to increase vaccination and has developed guidance for this with the Tailoring Immunization Programmes (TIP) approach.^5^^,^^6^

An ongoing measles outbreak in Romania causing 17 918 cases and 59 deaths in 2016–18,^7^ indicates sub-optimal vaccination. More evidence is needed to understand which population groups are affected and their reasons for sub-optimal vaccination. Vaccination records, individual recollection of vaccination status or stated intention to vaccinate may not always be accurate, so a measles outbreak provides an opportunity to identify and approach affected individuals. A study was conducted with three aims: (i) clarify who was affected by the measles outbreak, (ii) identify their barriers and drivers to vaccination and (iii) explore whether these factors varied by population group.

## Methods

The study included a review of outbreak surveillance data and survey with caregivers. Ethical approval was obtained by an ad hoc committee—academics from Departments of Public Health, Family Medicine and Psychology from University of Medicine Bucharest and BabesBolyai University of Cluj-Napoca.

### Review of outbreak surveillance data

The characteristics (age, geography, measles vaccination status) of all confirmed measles cases (from outbreak start late-January 2016 to mid-June 2017) were explored using national surveillance data recording all probable and confirmed measles cases using reports from district public health authorities.^8^ Measles deaths were identified from hospital reporting to public health districts and categorized based on the WHO case definition.^9^

### Survey with caregivers

A questionnaire was administered via telephone using the computer-assisted telephone interviewing method. Out of a total of 4187 confirmed/probable measles cases during January 2016–May 2017, aged 13 months to 18 years at the time of onset of measles, a random sample of 704 was selected, who were representative of the target population (county, gender and age), to allow estimation within at least a 3.7% margin of error. Ineligible cases (no contact details or consent) were replaced by cases matching location, gender and age. The response rate was 74%. This resulted in a representative sample of 520 out of 4187 measles cases (equating to 12%). Children below 13 months were excluded as non-vaccination of this group is in line with the national vaccination schedule. The selected cases were contacted by local health authorities and family doctors obtaining study consent and contact details. Characteristics of respondents and their child with measles were recorded: caregiver relationship to child, education, household income, ethnicity (majority vs. minority population), community setting (rural vs. urban), number of children; child age when contracted measles, disease classification, measles vaccination status and mobility. These characteristics were defined based on local assumptions and international evidence of determinants to vaccination.^10^ Mobility was defined as having lived in more than one place. Minority population was defined as ethnic minorities, such as Roma, Hungarians, Turkish, Hungarian Gypsies and others.^11^ Identification of minority/majority population was done using respondents’ addresses.

The questionnaire was designed using the WHO TIP approach,^5^^,^^6^ building on the COM-B model,^12^ identifying necessary conditions for behaviour change: capability (individual ability to enact the behaviour), physical and social opportunity (external physical or social environments that enable or inhibit the behaviour) and motivation (individual mechanisms that enact or inhibit behaviour) (Supplementary figure S1). Using a behaviour change model can ensure a comprehensive and systematic approach.^4^

All data were summarized descriptively for respondent characteristics and for each set of statements (capability, opportunity, motivation). Continuous measures were reported as means and standard deviations and for categorical data as counts and percentages.

To explore assumptions for different population groups, associations between responses to 11 pre-selected key statements and two characteristics (ethnicity, education) were compared using a *χ*^2^ test or Fisher’s exact test as appropriate. The ‘do not know/no responses’ option was excluded from the analyses. Exploring association with household income was not possible because 42% of respondents did not provide this information.

To quantify which characteristics (ethnicity, education, community setting, mobility) were associated with negative responses to nine pre-selected key statements, logistic regression models or multinomial logistic regression models were used depending on the comparisons. Odds ratios or relative risk ratios and corresponding 95% confidence intervals were obtained from the models.

## Results

### Characteristics of the affected population

#### Review of outbreak surveillance data

Most measles cases had received no (97%) or one dose (2%) of measles vaccination and lived in communities with suboptimal MMR vaccination coverage in 2015. In one district, coverage for the second dose of measles vaccine was just 29.7%. Particularly, affected districts were Arad (first dose: 67.9%, second dose: 47.7%), Caras-Severin (78.9%, 29.7%), Cluj (87.8%, 53.1%) and Timis (67.1%, 47.6%).^13^ The most affected age group was under 1 year, followed by 1–4 years and 5–9 years (Supplementary figure S2). In all, 1157 of 6743 cases were 15 years or older.

Of 6743 measles cases, there were 30 deaths, of which 13 children under 1 year; nine 1-years-olds, seven 2-to-17-year-olds and one 27-year-old. A measles mortality study in 2019 showed that key risk factors associated with measles-related death in 2016–18 included chronic respiratory illnesses and malnutrition.^14^

#### Survey with caregivers

Of 704 caregivers, 520 (74%) surveys were completed. Respondent characteristics are presented in table 1. The majority was the child’s mother (*n* = 366, 70%). Over 40% (*n* = 204) reported low levels of education (no education, primary education). There was an even split in ethnicity (minority population: *n* = 261, 50%, majority population: *n* = 259, 50%). Nearly 60% (*n* = 299) lived in rural locations. Since low/no education represent only 8% of the population in Romania, ethnic minorities represent an estimated 7% and rural populations represent 46%,^11^ this indicates a considerable over-representation of low education and ethnic minorities and an over-representation of rural population among measles cases.

**Table 1:** Description of survey respondents and their children (measles cases)

Characteristics	*N* (%)
Respondent’s relationship to child	
Mother	366 (70.4)
Father	117 (22.5)
Other	37 (7.1)
Education	
No education	46 (9.3)
Primary education	158 (31.9)
Secondary education	210 (42.4)
Tertiary education	81 (16.4)
Do not know/no response	25 (4.8)
Household income	
No income	2 (0.7)
Low (<1900)	138 (45.7)
Middle (2000–4999)	119 (39.4)
High (>5000)	43 (14.2)
Do not know/no response	218 (42.0)
Ethnicity^a^	
Ethnic minority	261 (50.2)
Ethnic majority	259 (49.8)
Community setting	
Urban	221 (42.5)
Rural	299 (57.5)
Number of children in household	
Mean (SD)	5.3 (2.2)
Median (min, max)	5 (2 to 20)
Do not know/no response	26 (5.0)
Which child contracted measles	
Mean (SD)	2.7 (1.8)
Median (min, max)	2 (1–13)
Do not know/no response	42 (8.0)
Child’s gender	
Female	253 (48.7)
Male	267 (51.4)
How old was child when contracted measles	
Mean (SD)	4.7 (4.3)
Median (min, max)	3 (1, 18)
Classification	
Confirmed	232 (44.6)
Possibly	1 (0.2)
Probably	287 (55.2)
Vaccination status (routine data)	
0 dose	460 (88.5)
1 dose	47 (9.0)
2 doses	13 (2.5)
How many doses (self-reported)	
0 dose	161 (31.0)
1 dose	354 (68.1)
Do not know	5 (1.0)
Child lived in the same community/village all life	
Yes	461 (88.7)
No	58 (11.2)
Do not know/no response	1 (0.2)
Child has lived in another place in Romania	
Yes	49 (9.4)
No	470 (90.4)
Do not know/no response	1 (0.2)
Child has lived abroad	
Yes	10 (1.9)
No	509 (97.9)
Do not know/no response	1 (0.2)

a Assumed ethnicity.

The mean number of children in the family was five, and the child who contracted measles was, usually, the second child (median = 2). There were approximately equal numbers of boys (*n* = 267, 51%) and girls (*n* = 253, 49%). Approximately one-third (31%, *n* = 161) reported having no dose of measles vaccine and 68% (*n* = 354) reported one dose, i.e. not in correspondence with routinely collected data which indicated that the vast majority of measles cases had not received any measles vaccination (Supplementary table S1). The majority (*n* = 461, 89%) indicated that their child had lived in the same community/village all their life.

#### Physical opportunity

Responses to 16 physical opportunity statements are presented in table 2.

**Table 2:** Summary of responses to physical/social opportunity, capability and motivation statements

Statement	Yes, *N* (%)	No, *N* (%)	Do not know/ no response, *N* (%)
Physical opportunity			
My child is registered with a family doctor	498 (95.8)	–	22 (4.2)
The health facility in general provides a high-quality service	479 (92.1)	10 (1.9)	31 (6.0)
The location of the health clinic is convenient to me	473 (91.0)	36 (6.9)	11 (2.1)
I feel welcome at the health facility	495 (95.2)	9 (1.7)	16 (3.1)
My children feel welcome at the health facility	475 (91.4)	8 (1.5)	37 (7.1)
The waiting area of the health facility is appropriate to my needs	487 (93.7)	14 (2.7)	19 (3.7)
Vaccines are provided at a convenient time of day for me	452 (86.9)	6 (1.2)	62 (11.9)
It is easy for me to get an appointment for vaccination	430 (82.7)	22 (4.2)	68 (13.1)
The waiting time at the health facility is appropriate	395 (76.0)	93 (17.9)	32 (6.2)
Measles vaccination is free of charge for my child	419 (80.6)	–	101 (19.4)
The measles vaccine was available when my child needed it	281 (54.4)	73 (14.0)	166 (31.9)
When I am living in other places my child(ren) get vaccinated in the place where we are	437 (84.0)	7 (1.4)	76 (14.6)
Someone from the doctors’ clinic informed me the last time it was time for my child’s vaccination	302 (58.1)	154 (29.6)	64 (12.3)
I am satisfied with the information I receive from my family doctor about vaccination	413 (79.4)	61 (11.7)	46 (8.9)
My family doctor provided clear information about vaccination	409 (78.7)	77 (14.8)	34 (6.5)
My family doctor provided clear information about potential side-effects	358 (68.9)	126 (24.2)	36 (6.9)
Social opportunity			
My family doctor advised me to vaccinate my child against measles	425 (81.7)	37 (7.1)	58 (11.2)
My family doctor advised me NOT to vaccinate my child against measles	9 (1.7)	460 (88.5)	51 (9.8)
Another doctor (other than my family doctor) advised me to vaccinate my child against measles	185 (35.6)	181 (34.8)	154 (29.6)
Another doctor (other than my family doctor) advised me NOT to vaccinate my child against measles	20 (3.9)	353 (67.9)	147 (28.3)
Media (TV, radio, newspapers) generally support vaccination	391 (75.2)	21 (4.0)	108 (20.8)
Most people who are important to me think that children should get vaccinated	416 (80.0)	49 (9.4)	55 (10.6)
Most people with young children in my community vaccinate their children	358 (68.9)	42 (8.1)	120 (23.1)
Capability			
I know where to go for vaccination of my child	457 (87.9)	63 (12.1)	–
When I am living in another place, I know where to go for vaccination	449 (86.4)	16 (3.1)	55 (10.6)
I know where to go for information about vaccination	368 (70.8)	152 (29.2)	–
I know when it is time for my child’s next vaccination	241 (46.4)	279 (53.7)	–
Motivation			
I believe that vaccines are generally safe for my child	371 (71.4)	59 (11.4)	90 (17.3)
The potential risk of vaccine side-effects is small	330 (63.5)	44 (8.5)	146 (28.1)
Measles is a potentially serious disease which can cause harm to my child	480 (92.3)	5 (1.0)	35 (6.7)
Vaccination is important for my child to have a healthy life	458 (88.1)	14 (2.7)	48 (9.2)
Vaccination is important to prevent spread of disease in my community	465 (89.4)	13 (2.5)	42 (8.1)
I intend to vaccinate my children according to the national schedule	410 (78.9)	37 (7.1)	73 (14.0)

The majority (*n* = 498, 96%) reported that their child is registered with a family doctor. Most indicated that the health facility provides high-quality service (*n* = 479, 92%), location is convenient (*n* = 473, 91%), they feel welcome (*n* = 495, 95%) as do their children (*n* = 475, 91%) and the waiting area is appropriate (*n* = 487, 94%). Many considered that the times when vaccines are provided are convenient (452, 87%); and it is easy to get an appointment for vaccinations (*n* = 430, 83%). Less respondents viewed the waiting time as appropriate (*n* = 395, 76%). Respondents with lower levels of education were less likely to think that the health facility provides high-quality service compared with higher education levels (table 3). There was no evidence of variation by ethnicity or education levels for statements on feeling welcome, waiting area, time of day or ease of getting an appointment (table 3).

**Table 3:** Associations between key statements and ethnicity and education

Statements	Ethnicity^a^*N* (%)			Education *N* (%)				
	Ethnic minority^b^	Ethnic majority^b^	*P* value	No education	Primary	Secondary	Tertiary	*P* value
Physical opportunity								
The health facility in general provides a high-quality service	248 (98.0)	241 (97.9)	1.000	42 (97.7)	146 (95.4)	197 (99.5)	76 (98.7)	0.050
I feel welcome at the health facility	234 (98.7)	218 (98.6)	1.000	45 (100.0)	141 (100.0)	183 (96.8)	68 (100.0)	0.078
The waiting area of the health facility is appropriate to my needs	251 (98.1)	244 (98.4)	1.000	37 (80.4)	121 (80.7)	158 (81.4)	66 (81.5)	0.997
Vaccines are provided at a convenient time of day for me	240 (93.8)	233 (92.1)	0.466	43 (95.6)	146 (93.0)	192 (92.3)	74 (92.5)	0.894
It is easy for me to get an appointment for vaccination	227 (95.4)	203 (94.9)	0.798	35 (89.7)	133 (95.0)	178 (95.7)	70 (95.9)	0.471
I am satisfied with the information I receive from my family doctor about vaccination	212 (87.6)	201 (86.6)	0.754	35 (83.3)	128 (88.9)	171 (86.8)	66 (88.0)	0.801
Social opportunity								
My family doctor advised me to vaccinate my child against measles	200 (90.1)	225 (93.8)	0.148	39 (95.1)	135 (93.8)	172 (91.5)	65 (89.0)	0.552
Most people who are important to me think that children should get vaccinated	211 (91.3)	205 (87.6)	0.190	37 (97.4)	132 (91.7)	173 (90.6)	60 (79.0)	0.006
Motivation								
The potential risk of vaccine side-effects is small	158 (87.8)	172 (88.7)	0.791	29 (90.6)	108 (91.5)	143 (90.5)	39 (72.2)	0.002
Vaccination is important for my child to have a healthy life	227 (97.0)	231 (97.1)	0.974	41 (100.0)	148 (98.7)	192 (96.0)	63 (95.5)	0.255
I intend to vaccinate my children according to the national schedule	213 (93.8)	197 (89.6)	0.100	45 (100.0)	126 (91.3)	170 (92.9)	55 (83.3)	0.015

Analyses excluded do not know/no response option.

a Assumed ethnicity.

b Analysis included do not know/no response as there were zero no responses reported for this question.

Most (*n* = 419, 81%) agreed that measles vaccination is provided free of charge to their child and that when living in other places their child(ren) still gets vaccinated (*n* = 437, 84%); although 19% and 15%, respectively, did not know/did not respond. Respondents who reported that their child had lived in different places and those with primary level education (compared with tertiary) were twice as likely as those whose child had lived in the same place to not know/not respond that measles vaccination is free (table 4). There was no association with ethnicity or rural/urban setting for this statement (table 4).

**Table 4:** Predictors of a negative response to key physical/social opportunity, capability and motivation statements

Statement	No vs. yes			No vs. do not know		
	Type	Estimate (95% CI)	*P* value	Type	Estimate (95% CI)	*P* value
Physical opportunity						
Measles vaccination is free of charge for my child						
Ethnicity (minority/majority)^a^	OR	1.26 (0.81–1.94)	0.299			
Education^a^						
No education	OR	1.60 (0.76–4.05)	0.324			
Primary	OR	2.01 (0.99–4.09)	0.053			
Secondary	OR	0.88 (0.43–1.84)	0.742			
Living in the same community	OR	2.05 (1.12–3.75)	0.020			
Community (urban/rural)	OR	1.29 (0.83–1.99)	0.260			
The measles vaccine was available when my child needed it						
Ethnicity (Minority/Majority)^a^	RRR	1.07 (0.64–1.79)	0.804	RRR	0.84 (0.49–1.46)	0.540
Education^b^						
Primary	RRR	0.93 (0.33–2.55)	0.881	RRR	1.19 (0.41–3.44)	0.748
Secondary	RRR	1.02 (0.38–2.71)	0.975	RRR	1.42 (0.51–3.95)	0.506
Tertiary	RRR	1.13 (0.38–3.37)	0.823	RRR	1.53 (0.49–4.85)	0.465
Living in the same community	RRR	0.45 (0.15–1.32)	0.146	RRR	0.38 (0.13–1.14)	0.083
Community (urban/rural)	RRR	0.72 (0.42–1.32)	0.230	RRR	0.70 (0.40–1.24)	0.228
Someone from the doctors’ clinic informed me the last time it was time for my child’s vaccination						
Ethnicity (minority/majority)^a^	RRR	1.50 (1.02–2.22)	0.040	RRR	0.67 (0.37–1.23)	0.197
Education^b^						
Primary	RRR	3.39 (1.41–8.14)	0.006	RRR	2.81 (0.71–11.13)	0.141
Secondary	RRR	2.60 (1.09–6.18)	0.030	RRR	1.59 (0.43–5.94)	0.490
Tertiary	RRR	2.22 (0.84–5.84)	0.106	RRR	0.71 (0.18–2.88)	0.636
Community (urban/rural)	RRR	1.27 (0.85–1.87)	0.240	RRR	0.72 (0.40–1.29)	0.263
My family doctor provided clear information about vaccination						
Ethnicity (minority/majority)^a^	RRR	1.26 (0.84–1.92)	0.256	RRR	0.69 (0.45–1.06)	0.091
Education^b^						
Primary	RRR	2.33 (1.06–5.12)	0.035	RRR	1.03 (0.42–2.49)	0.950
Secondary	RRR	1.63 (0.77–3.46)	0.205	RRR	1.23 (0.51–2.95)	0.644
Tertiary	RRR	1.15 (0.47–2.77)	0.761	RRR	0.61 (0.23–1.63)	0.327
Community (urban/rural)	RRR	1.11 (0.73–1.68)	0.620	RRR	1.03 (0.67–1.59)	0.884
Social opportunity						
Most people with young children in my community vaccinate their children						
Ethnicity (minority/majority)^a^	RRR	0.84 (0.44–1.61)	0.607	RRR	0.75 (0.37–1.51)	0.419
Education^b^						
Primary	RRR	4.64 (0.59–36.26)	0.144	RRR	8.23 (1.00–67.77)	0.050
Secondary	RRR	3.33 (0.43–26.12)	0.252	RRR	4.80 (0.59–39.24)	0.143
Tertiary	RRR	2.95 (0.34–25.62)	0.327	RRR	6.43 (0.69–60.31)	0.103
Community (urban/rural)	RRR	1.25 (0.66–2.37)	0.492	RRR	1.93 (0.94–3.93)	0.071
Capability						
I know where to go for vaccination of my child						
Ethnicity (minority/majority)^a^	OR	1.89 (1.10–3.26)	0.022			
Education^b^						
No education	OR	0.26 (0.06–1.22)	0.088			
Primary	OR	0.98 (0.46–2.09)	0.957			
Secondary	OR	0.47 (0.21–1.05)	0.067			
Community (urban/rural)	OR	0.94 (0.55–1.61)	0.833			
I know where to go for information about vaccination						
Ethnicity (minority/majority)^a^	OR	1.47 (1.00–2.15)	0.048			
Education^b^						
No education	OR	3.37 (1.43–7.94)	0.005			
Primary	OR	3.25 (1.62–6.49)	0.001			
Secondary	OR	1.99 (1.00–3.96)	0.049			
Living in the same community	OR	1.99 (1.14–3.48)	0.016			
Community (urban/rural)	OR	0.84 (0.57–1.23)	0.370			
I know when it is time for my child’s next vaccination						
Ethnicity (minority/majority)^a^	OR	1.32 (0.94–1.87)	0.112			
Education^b^						
No education	OR	1.39 (1.67–2.88)	0.381			
Primary	OR	1.11 (0.65–1.89)	0.709			
Secondary	OR	1.01 (0.61–1.69)	0.959			
Community (urban/rural)	OR	1.27 (0.89–1.80)	0.187			
Motivation						
I believe that vaccines are generally safe for my child						
Ethnicity (minority/majority)^a^	RRR	0.91 (0.52–1.57)	0.731	RRR	0.86 (0.45–1.67)	0.663
Education^b^						
Primary	RRR	0.79 (0.24–2.58)	0.692	RRR	1.86 (0.49–7.00)	0.360
Secondary	RRR	1.15 (0.37–3.57)	0.804	RRR	2.40 (0.69–8.40)	0.171
Tertiary	RRR	2.40 (0.73–7.85)	0.148	RRR	3.20 (0.84–12.12)	0.087
Community (urban/rural)	RRR	1.63 (0.94–2.83)	0.082	RRR	1.38 (0.72–2.67)	0.334

OR = odds ratio; RRR = relative risk ratio.

a Assumed ethnicity.

b Compared with no education.

Only half (*n* = 281, 54%) reported that that the measles vaccine was available when they needed it, nearly one third (*n* = 166, 32%) did not know/did not respond. There was little or no evidence of association between people responding that the vaccine was not available and ethnicity, education, mobility or rural/urban setting (table 4).

Only 58% (*n* = 302) reported they had received information from the doctor’s clinic about their child’s next vaccination. Minority population respondents were 1.5 times more likely than majority population respondents to report they had not been informed. There was no association with rural/urban setting (table 4).

Many (*n* = 409, 79%) reported their family doctor had provided clear information about vaccination. There was no association with ethnicity, secondary/tertiary education levels compared with no education and rural/urban setting (table 4). Only a quarter (*n* = 126, 24%) said that their family doctor had provided clear information about potential side effects.

#### Social opportunity

Responses to seven social opportunity statements are presented in table 2.

Most respondents (*n* = 425, 82%) indicated that their family doctor had advised them to vaccinate their child against measles (no evidence of variation across education levels or ethnicity, table 3). Few respondents stated that their family doctor (1%) or another doctor (4%) had advised against measles vaccination.

Three quarters of respondents stated that the media support vaccination (*n* = 351, 75%). Four out of five said people who are important to them think children should be vaccinated (*n* = 416, 80%). This varied across education levels (not ethnicity) (table 3). Those with tertiary education (*n* = 60, 79%) were less likely to agree compared with those with secondary (*n* = 173, 90.6%) or primary (*n* = 132, 91.7%) education. Slightly fewer respondents (*n* = 358, 69%) reported that most people in their community vaccinate their children (table 4).

#### Capability

Responses to four capability statements are presented in table 2.

The majority reported that they know where to go for vaccination of their child (*n* = 457, 88%) even when living in another place (*n* = 449, 86.4%). The minority population was less likely than the majority population to know where to go. There was no evidence of variation across education levels or rural/urban setting (table 4).

Less than three quarters (*n* = 368, 71%) knew where to go for information about vaccination. Minority population respondents were 1.5 times as likely as majority population not to know where to go for information. Those with no education/primary education were three times as likely not to know where to go compared with those with tertiary education. Those with secondary education were twice as likely not to know compared with those with tertiary education. Respondents whose child had lived in different communities were twice as likely as those whose child had lived in the same community not to know. There was no association with rural/urban setting (table 4).

Less than half (*n* = 241, 46%) knew when it was time for their child’s next vaccination. There was no evidence of an association between ethnicity, education and rural/urban setting (table 4).

#### Motivation

Responses to six motivation statements are presented in table 2.

Notably, 17% (*n* = 90) did not know/did not respond or disagreed (*n* = 59, 11%) with the statement that vaccines are generally safe for their child. There was no evidence of variation across ethnicity, education levels or rural/urban setting (table 4).

Over a quarter (28%, *n* = 146) did not know/did not respond or disagreed (*n* = 44, 9%) with the statement that the potential risk of vaccine side effects is small. Over one fifth did not know/did not respond (*n* = 73, 14%) or did not intend (*n* = 37, 7%) to vaccinate their child according to the national schedule.

Respondents with tertiary education levels were less likely to think that the potential risk of vaccine side effects is small or to vaccinate their child according to the national schedule compared with those with lower education levels; there was no evidence of variation by ethnicity for either statement (table 3).

The majority thought that measles is a potentially serious disease which can cause harm (*n* = 480, 92%), vaccination is important for their child to have a healthy life (*n* = 458, 88%) and vaccination is important to prevent spread of disease in their community (*n* = 465, 89%). There was no evidence of an association between ethnicity or education for the belief that vaccination is important for their child to have a healthy life (table 3).

## Discussion

There are many assumptions but little evidence of the reasons behind sub-optimal vaccination uptake in Romania. This is the first study to use national data to identify the characteristics of measles cases and their barriers and drivers to vaccination. Using a behaviour change model^12^ ensured a comprehensive, theory-informed approach.

Some limitations should be acknowledged. With 42% of respondents not indicating household income, it was impossible to explore the influence of this. Another limitation related to identifying majority/minority populations. Categorizing people by ethnicity is culturally inappropriate in Romania, hence surveillance data does not identify ethnicity. Identification of minority/majority populations was done manually after data collection using respondents’ addresses rather than within the sampling procedure or using respondents’ self-identification. Ethnic disintegration is significant in Romania, so living area is a strong predictor of ethnicity.^11^ Still, this categorization may be confounded by subjective interpretation. While it is a study strength that we include only parents of sub-optimally immunized children, it is a limitation that we reach them when their response might be biased by their child having already contracted measles and needing them to recollect past experiences or perceptions. Furthermore, given this is a cross sectional survey we cannot infer causation. Finally, as it may be assumed that caregivers with the least interaction with the health system (e.g. unregistered) may not have taken part in the survey, we cannot claim that our findings represent their views.

The surveillance data indicating that children with measles were mostly unvaccinated and living in low-coverage areas confirms that the outbreak is due to suboptimal vaccination uptake, and that geographic areas with pockets of susceptible populations need to be targeted to avoid future outbreaks.

The survey findings were generally positive about health services. Most caregivers felt welcome at the health facility, were content with their location, quality of services, appointment systems and waiting areas. These factors can affect vaccination decisions and lead to vaccine hesitancy,^15^ but appear to be less of a concern. Less well reviewed was waiting time.

Some responses about access to vaccination were concerning. Only half of caregivers believed the measles vaccine to be available when needed. Indeed, vaccine shortages have been experienced in Romania. A similar proportion did not know when their child’s next vaccination was due and had not been told by their family doctor, particularly evident for minority population respondents and those with middle-level education. There is good international evidence that reminder systems can improve vaccination coverage.^16^ An electronic registry has recently been introduced in Romania, and text message reminders are being piloted; however, a structured reminder system is not being introduced at this point. Many caregivers who were mobile or less educated did not know that the measles vaccine is provided free of charge. This misperception was a surprise for health authorities, and the reasons behind it should be explored and addressed.

Respondents’ beliefs about the benefits of vaccination and the risks of measles were generally supportive of future vaccination. However, concerns about vaccine safety were evident, particularly among the most educated who were also less likely to perceive that people who are important to them supported vaccination. Such concerns are critical for vaccine decisions^15^^,^^17^ and can be a reason for vaccine refusal. Whilst three quarters knew where to go for information about vaccination; the minority population group, those with less education or who were mobile were less informed. Only a quarter reported receiving clear information from their doctor about side effects. The family doctor is central in shaping people’s vaccination behaviour^18^^,^^19^ and there is a need to continuously build their skills to advise parents, respond to safety concerns and questions and provide the necessary information and reassurance.^20^^,^^21^

Prior to this study, concerns had been raised that some doctors’ advice may be affected by their own hesitancy to vaccination; this was neither confirmed nor rejected. Less than 4% of caregivers indicated having been advised against vaccination by a doctor; however, any possible subtle hesitancy, e.g. in the form of covert recommendations against vaccination or application of false contraindications are not captured here.

The study found that barriers to vaccination in Romania are complex and relate to all factors of the COM-B model.^12^ However, factors varied by population group. Disadvantaged groups such as minority, mobile and low education groups were highly over-represented among measles cases, and malnutrition was a risk factor associated with measles-related deaths. For these groups, capability and physical opportunity barriers to vaccination were particularly evident. For those with higher education levels, the challenges related more to motivation and social opportunity factors. These findings are consistent with other countries in Europe and further afield.^15^^,^^22^^,^^23^

No one action can turn this situation around; a multipronged strategy is needed tailored to the needs of different population groups. Studies elsewhere confirm an association between low immunization uptake and social determinants which are associated with other health inequities, including low levels of parental education and income.^10^ Still, efforts can be done to ensure an equitable vaccination programme. Other interventions are needed to build trust and social forms in favour of vaccination among well-educated caregivers.

In this complex context, the findings led to recommendations for actions to avoid a future outbreak, including improved service provision and capacity building of vaccination providers, engaging trusted stakeholders in enhancing confidence in vaccination and strengthening vaccine supply. The study also exposed a need for more in-depth insights, including into the quality and convenience of services, and so has informed the design of an observation study with family practices serving vulnerable communities, which is now being completed.

## Conclusion

Identifying the characteristics of the population affected by the measles outbreak and their barriers to vaccination allowed to inform actions to avoid future outbreaks, tailored to the needs of different population groups.

## Supplementary Material

ckaa079_Supplementary_Data
