# Supplementary material for: Measles outbreak in Romania: understanding factors related to suboptimal vaccination uptake
Source: Eur J Public Health. 2020 May 27;30(5):986–92. doi: 10.1093/eurpub/ckaa079 (PMC7536253; doi:10.1093/eurpub/ckaa079)
Supplement: ckaa079_Supplementary_Data [file ckaa079_supplementary_data.doc]

**eTable 1. Measles cases by MMR vaccination status (only in online version)**

| No MMR dosies | Age group (years) | | | | | | | | | | |
| --- | --- | --- | --- | --- | --- | --- | --- | --- | --- | --- | --- |
|  | <1 | 1-4 | 5-9 | 10-14 | 15-19 | 20-24 | 25-29 | 30-34 | 35-39 | >40 | Total |
| 0 | 1306 | 2558 | 1097 | 456 | 312 | 201 | 195 | 140 | 149 | 109 | 6523 |
| 1 | 0 | 91 | 32 | 7 | 9 | 3 | 3 | 1 | 6 | 0 | 152 |
| 2 | 0 | 1 | 15 | 16 | 12 | 8 | 7 | 2 | 0 | 0 | 61 |
| Unknown | 0 | 5 | 2 | 0 | 0 | 0 | 0 | 0 | 0 | 0 | 7 |
| Total | 1306 | 2655 | 1146 | 479 | 333 | 212 | 205 | 143 | 155 | 109 | 6743 |

**eFigure 1. Factors explored to understand barriers and drivers to vaccination (adapted from 7) (only in online version)**


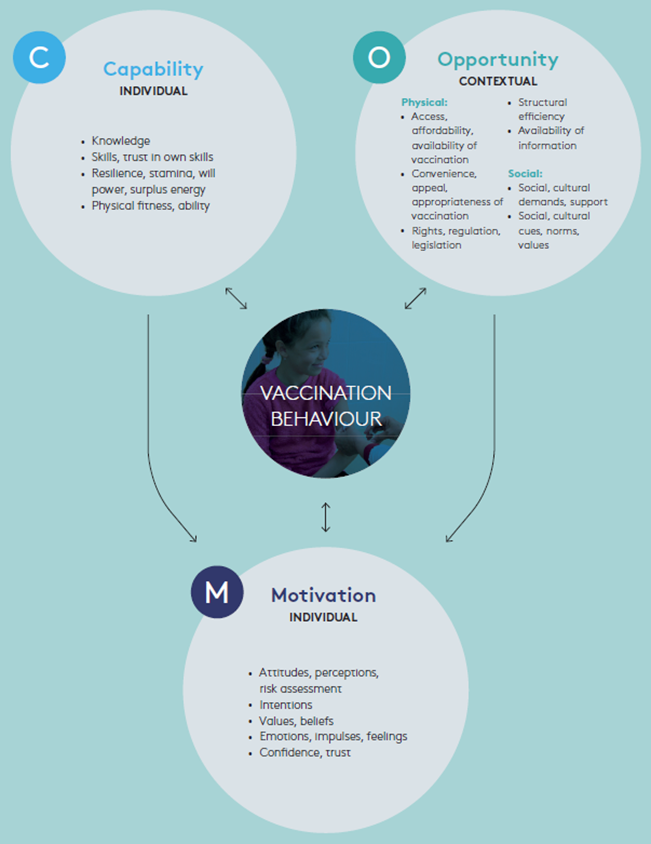


**eFigure 2. Incidence rate of measles cases in Romania, by age groups, during the initial 18 months of the outbreak (January 2016-June 2017) (N=6743) (only in online version)**
